# Cosmetotextiles with Gallic Acid: Skin Reservoir Effect

**DOI:** 10.1155/2013/456248

**Published:** 2013-04-11

**Authors:** Meritxell Martí, Cristina Alonso, Vanessa Martínez, Manel Lis, Alfons de la Maza, José L. Parra, Luisa Coderch

**Affiliations:** ^1^Advanced Chemical Institute of Catalonia, (IQAC-CSIC), Jordi Girona 18-26, 08034 Barcelona, Spain; ^2^Terrassa School of Engineering (EET-UPC), Colom 1, 08222 Terrassa, Spain

## Abstract

The antioxidant gallic acid (GA) has been incorporated into cotton (CO) and polyamide (PA) through two different vehicles, that is, liposomes and mixed micelles, and their respective absorption/desorption processes have been studied. Moreover, *in vitro* percutaneous absorption tests of different cosmetotextiles have been performed to demonstrate antioxidant penetration within the layers of the skin. When GA was embedded into the cosmetotextiles, it always promoted a reservoir effect that was much more marked than that observed for polyamide. Similar penetration was observed in the textiles treated with GA in mixed micelles or liposomes in such compartments of the skin as the stratum corneum, epidermis, and even the dermis. GA was detected in receptor fluid only when CO was treated with MM. This methodology may be useful in verifying how encapsulated substances incorporated into textile materials penetrate human skin. Indeed, such materials can be considered strategic delivery systems that release a given active compound into the skin at specific doses.

## 1. Introduction

Cosmetotextiles are garments designed to contact the skin with the aim of transferring active substances useful for cosmetic purposes, particularly to combat ageing effects [1–4]. In fact, there are already several textile products on the market that claim to have certain properties that are usually found in pharmaceuticals or cosmetics [3], such as moisturising, slimming, energising, refreshing, relaxing, vitalizing, or UV-protecting properties, or are simply perfume. There is a real need to develop test methods to demonstrate and verify the effectiveness and durability of these claimed properties [5]. 

Encapsulation is one of the techniques used to apply such substances to textiles [6]. Liposomes are biocompatible, biodegradable, and nontoxic artificial vesicles formed by lipids that can encapsulate many compounds (hydrophilic, hydrophobic, and amphiphilic) for application to textiles. Moreover, liposomes have been the subject of numerous studies because of their importance as microencapsulation devices for drug delivery and their applications in cosmetics [7–10]. In fact, one way of enhancing drugs' skin penetration is the use of vesicular systems or liposomes [9, 10]. In this study, a new strategy to enhance the delivery of an active agent from a textile to the skin using mixed micelles (MMs) was investigated. The micelles are composed of a lipid and a surfactant and are capable of transforming into liposomes when the surfactant is eliminated by simple dilution with water. The potential ability of the MM to be structured as liposomes in textile fabrics by dilution in water was investigated [11, 12].

Antioxidants are substances used as natural resources to regulate processes considered external threats to the body, preventing oxidative stress. One of the body's defence systems is the generation of endogenous antioxidants. The body also incorporates exogenous antioxidants into the diet. It has been demonstrated that when topically applied, these exogenous antioxidants can diminish the effects of free radicals using defence mechanisms similar to those of endogenous antioxidants [13, 14]. The encapsulation of antioxidants in liposomes improves their therapeutic potential against oxidant-induced tissue injuries, because liposomes facilitate intracellular delivery [15]. In this regard, textiles containing antioxidants might have diffusion characteristics similar to those of transdermal relize patches used in the field of pharmaceuticals. In this study, the phenolic acid GA was used as an active principle for its anti-inflammatory, antifungal, and antiviral properties and the antioxidant protection it provides to our cells against free radicals [16]. The ability of GA to serve as a reliable chemical tracer and its beneficial effects lend support to its incorporation into a textile designed to be used in contact with the skin.

In the present work, the antioxidant GA has been incorporated into CO and PA through two different vehicles, Lip and MM, and their respective absorption/desorption behaviours have been studied [17]. The aim of this work was to determine GA penetration from different biofunctional textiles within the layers of the skin using a specific *in vitro* percutaneous absorption method [18].

## 2. Methodology

### 2.1. Materials

The standard fabrics used were plain cotton fabric (CO) (Bleached Desized Cotton Print Cloth, Style 400 ISO 105-F02) and spun polyamide fabric (PA) (Style 361, ISO 105-F03). Liposomes were prepared using commercial lipids (phospholipids) Emulmetik 900 (Lucas Meyer GMbH, France), and mixed micelles were prepared using the same lipids and the surfactant Oramix CG 110 (Caprylyl/Capryl Glucoside) (Seppic Italia Srl, Italy). The antioxidant active agent gallic acid (GA) (Sigma-Aldrich) was employed. All chemicals used were of analytical grade. Methanol (HPLC-grade) and distilled water were used for high-performance liquid chromatography analysis with UV detection (HPLC-UV). Methanol (Carlo Erba, France) was used to extract GA from the textiles.

### 2.2. Liposome/Mixed Micelle Preparation

Emulmetik 900 is a waxlike soybean lecithin emulsifier with an enriched content of phosphatidylcholine for use in the cosmetic industry and was employed for Lip and MM formation. Lips containing 4% Emulmetik 900 (PC) and 2% gallic acid (GA) were prepared using the thin-film hydration method reported elsewhere [19]. PC (4 g) solubilised in chloroform was dried. The lipid film was dispersed in 100 mL of a 2% GA aqueous solution, and multilamellar vesicles (MLV) were obtained. MMs (30% surfactant, 4% PC, and 2% GA) were prepared by solubilising all compounds in distilled water; solubilisation was performed by gently shaking until clear solutions were obtained. All activities took place at room temperature. 

Dynamic Light Scattering (DLS) (Zetasizer Nano ZS ZEN3600; Malvern Instruments Ltd., Malvern, Worcestershire, UK) was used to determine the size distribution and polydispersity index of the Lip and MM. A noninvasive backscattering technique was used to minimise multiple scattering effects without the need to dilute the samples. The measurement was performed at room temperature with polystyrene cells (Ref 67.754 Sarstedt). The detection of the light scattered was performed at an angle of 173°. Each sample was measured in triplicate. The data were interpreted by correlating the particle size distribution with the intensity of light scattered. All data were collected and analysed using the software programme Dispersion Technology Software (DTS) provided by Malvern Instruments Ltd.

To quantify the GA entrapped in the vesicles, a Lip formulation was precipitated and separated from the supernatant by centrifugation at 14000 RPM for 15 minutes using a Centrifuge 5415-Eppendorf (Germany). After separation, the supernatant was retained. The initial liposome dispersion and the supernatant were diluted in isopropanol/water 1/1 and read spectrophotometrically at 269 nm (GA maximum absorption) using a Cary BIO300 spectrophotometer. The efficacy entrapment percentage of GA in the Lip was determined by taking into account the amount of the active principle present in the entire liposome dispersion (GA_ Lip _), as well as in the supernatant (GA_ supernatant _) (see (1)), using a GA calibration curve:

(1) $$\begin{array}{c} \%E=\frac{\text{G}{\text{A}}_{\text{ Lip }}-\text{G}{\text{A}}_{\text{ supernatant }}}{\text{G}{\text{A}}_{\text{ Lip }}}\times 100. \end{array}$$

### 2.3. Textile Application: Absorption/Desorption Process

Lips and MMs containing GA were applied onto CO and PA fabrics in triplicate by bath exhaustion in a liquor ratio of 1/5 at 60°C for 60 min with manual stirring every 10 minutes. To quantify the amount of Lip or MM absorbed into the fabrics, the samples were weighed before and after application under 24 h standard ambient conditions (23 ± 2°C and 50 ± 5% relative humidity, ISO 554-1976). 

To study the desorption process, the treated fabrics were washed in three different water baths at room temperature; the samples were weighed before and after each washing with deionised water (1/5 bath ratio, 1/10 bath ratio, and 1/25 bath ratio, resp.) for 5 min with magnetic stirring under 24 h standard ambient conditions (23 ± 2°C and 50 ± 5% relative humidity, ISO 554-1976). Particle sizes were measured in the baths after the exhaustion treatment and in the baths after the first and third washings as described for the initial formulations.

### 2.4. *In Vitro* Percutaneous Absorption Experiments (Franz Diffusion Cells)

For these studies, pig skin was used from the unboiled backs of large white/Landrace pigs weighing 30–40 kg. The pig skin was provided by the Clínic Hospital of Barcelona, Spain. After excision, the skin was dermatomed to a thickness of approximately 500 ± 50 *μ*m with a Dermatome GA630 (Aesculap, Germany). Skin discs with a 2.5 cm inner diameter were prepared and fitted into static Franz-type diffusion cells.

**Figure 1:**
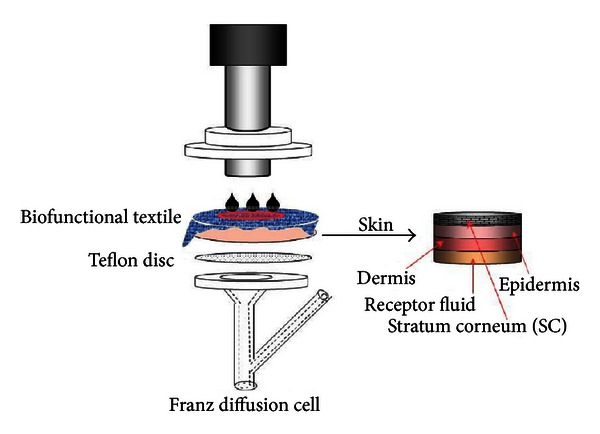
Diagram of *in vitro* percutaneous absorption experiments.

Skin absorption studies were initiated by applying 10 *μ*L of Lip or MM (approximately 70 *μ*g/cm^2^ GA) or by applying the fabrics treated with the same Lip or MM (containing approximately 150–250 *μ*g/cm^2^ GA) onto the skin surface. Between the textile and the skin, 20 *μ*L of distilled water was added to ensure close contact. A control skin disc (without product application on the skin surface) was used to rule out possible interferences in the analysis of GA by HPLC-UV. According to the OECD methodology [20], the skin penetration studies were performed for 24 h of close contact between the textile and the skin. To increase the contact pressure between the textile fabric and skin, permeation experiments were also carried out by placing a steel cylinder on the textile-skin substrate at a constant pressure in accordance with standard conditions (125 g/cm^2^) (ISO 105-E04, 1996) [21] (see Figure 1). 

After the exposure time, the receptor fluid was collected and brought to a volume of 5 mL in a volumetric flask. In the case of the formulations, the skin surface was washed with a specific solution (500 *μ*L SLES (sodium lauryl ether sulphate) (0.5%) and twice with 500 *μ*L distilled water) and dried with cotton swabs. In the case of the textiles, the fabrics were removed from the skin surface and collected together with the top of the cell. In both cases, after eliminating the excess GA from the skin surface, the stratum corneum of the skin was removed using adhesive tape (D-Squame, Cuderm Corporation, Dallas, TX, USA) applied under controlled pressure (80 g/cm^2^ for 5 sec). The epidermis was separated from the dermis after heating the skin to 80°C for five seconds.

GA was extracted from the different samples (surface excess, CO/PA or skin layers) using a methanol : water (50 : 50) solution agitated in an ultrasonic bath for 30 min at room temperature. The receptor fluids were directly analysed. After filtration on a Millex filter (0.22 *μ*m, Millipore, Bedford, MA, USA), the solutions containing GA were assessed by HPLC-UV. 

### 2.5. Gallic Acid Analytical Detection

The GA extracted from the different samples was determined by HPLC equipped with a UV-Vis detector as previously described [19]. The column used was a LiChroCART 125-4/LiChrosorb RP-18 (5 *μ*m) (Darmstadt, Germany). The mobile phase was 80% water/20% methanol flowed at a rate of 1 mL/min. The GA retention time was approximately 3.3 min. The area under curve was used to calculate the concentration of GA using external standards that showed linearity over a concentration range from 0.25 to 100 *μ*g/mL. Four textiles treated with the formulation without GA were studied as blank samples. This experimental methodology prevented any compound from possibly interfering with the analysis of the target substance. The analytical methodology was fully validated.

### 2.6. Data Treatment

Analysis of variance (ANOVA) was used to determine significant differences between percutaneous absorption values obtained from different samples (significant level accepted *P < 0.01) using the Statgraphics program.

## 3. Results

Two textiles, CO and PA, were chosen to compare the roles of two vehicles, two phospholipid structures (Lip and MM) composed of the same phospholipids but with a surfactant in the case of MM. Both vehicles were applied by bath exhaustion, as it was explained in Section 2 to study the absorption and desorption of GA.

The Lip formulation was prepared by thin-film hydration method, as described in Section 2, with 4% phospholipids (PC) and 2% GA in water. Polydisperse vesicle suspensions (0.7 PdI) with especially large multilamellar vesicles (MLV 700 nm) were formed [22]. Vesicles of this type may contain, on average, up to 10 bilayers [23]. The multilamellar structure allows for high encapsulation efficiency for both hydrophilic and hydrophobic substances, which can be localised not only in the central core of the vesicle but also in the aqueous interlamellar spaces or in the multiple lamellar spaces [24]. To quantify the GA entrapped in the vesicles, the Lip formulation was precipitated and separated from the supernatant by centrifugation. The amount of GA in the initial Lip solution and in the supernatant and the difference between them were evaluated as described in Section 2, yielding a fairly high entrapment efficacy of 31.9 ± 9.6%.

In MM, the two constituent PC and the surfactant agent are structured together in small micelles, giving rise to a transparent solution. However, the dilution of MM promotes the separation of the surfactant and the PC with the formation of liposomes. This results in a large increase in size, giving rise to a turbid solution [7]. The absorption of micelles by textiles could be maintained after washing because of the expected increase in the size of the vehicles inside the textile fibres, which could enhance the fixation of the micelles in textiles with less desorption, as occurs in the skin [25, 26]. 

The variation in the vehicle sizes with dilution for the two formulations is presented in Table 1. The Lip size decreases from approximately 700 to 400 nm with dilution, whereas the MMs have an initial size of 8 nm and increase to 55 nm for a dilution to 2.5% MM in water, as expected, due to liposome formation with dilution. The PDI remained unaltered by the dilution effect in both lipid structures. The Lip PDI (>0.600) was much higher than MM PDI (*≅*0.1), indicating a higher dispersion for the big structures. Besides, it has to be pointed out that the Lips were not extruded.

The active agents vehiculised in Lip (4% PC, and 2% GA = 6% dry product) and MM (30% surfactant, 4% PC and 2% GA = 36% dry product) were applied to the textile substrates, CO and PA, by bath exhaustion as described in Section 2. The initial and final percentages of dry product calculated by the weight difference between the dry initial fabric and dry fabric after bath exhaustion are shown in Table 2. 

When the MMs were applied to the fabrics by an exhaustion process, higher absorption than that in the Lip-treated fabrics was observed. Nevertheless, extremely high desorption was observed due to the Lip treatment of both textiles. It is important to note that PA absorbed much more MMs and Lips than CO. The interaction of lecithin with CO has been reported to occur mainly at the surface through a coating layer, whereas the interaction with PA occurs in the interior of the fibres [27].

The higher absorption of MM in CO and especially in PA could be due to the presence of 30% Oramix. The increase in particle size with dilution in the washing baths, which reached up to 50–100 nm, did not prevent desorption. A large amount of desorption occurs in MM-treated fabrics. The separation of Lip composed of phospholipids and micelles featuring Oramix could increase their affinity for water in the two textiles, favouring desorption. The desorption of Lip from the PA- and CO-treated fibres was approximately 50%, whereas the desorption of MM from the PA- and CO-treated fibres was 90%. The particle size of the lipid structures of Lip and MM was evaluated in the initial, after treatment, and after washing baths (Table 3) to determine the possible influence on product desorption.

A comparison of the results in Table 3 with those obtained for Lip and MM elution in Table 1 shows that in all the baths of CO and PA, the Lip exhibited a similar size of approximately 500 nm. In the initial baths, the MM presented very small sizes of approximately 7 nm. However, a size increase of up to 100–200 nm was already observed in the initial bath after the treatment as well as in the washing baths. The concentration of MM in the bath after textile treatment or after washing treatments is much lower than the one assayed in Table 1; the surfactant could have been removed in a high extent, and lipids could have been organised as vesicles higher in size. The extremely high absorption of MM could be due to the easy penetration of the small structures into fabrics. However, the increase in the size of these structures (see Table 3) did not prevent their exit from the fibres, and desorption was notable. This finding could be due to the higher permeability of textiles compared with human skin, which may explain why this effect was not observed [25, 26].

To study the penetration of active principles through the skin, an *in vitro* methodology based on percutaneous absorption is performed to demonstrate the delivery of an encapsulated principle from a textile to the different layers of the skin (stratum corneum, epidermis, or dermis).

The percutaneous absorption of the two formulations, Lip (2% GA, 4% PC) and MM (2% GA, 4% PC, and 30% Oramix CG 110), was evaluated, as were the CO and PA textiles impregnated with the same Lip or MM. The two formulations and the CO and PA textiles previously treated with the formulations were placed in contact with the skin discs as described in Section 2.

**Figure 2:**
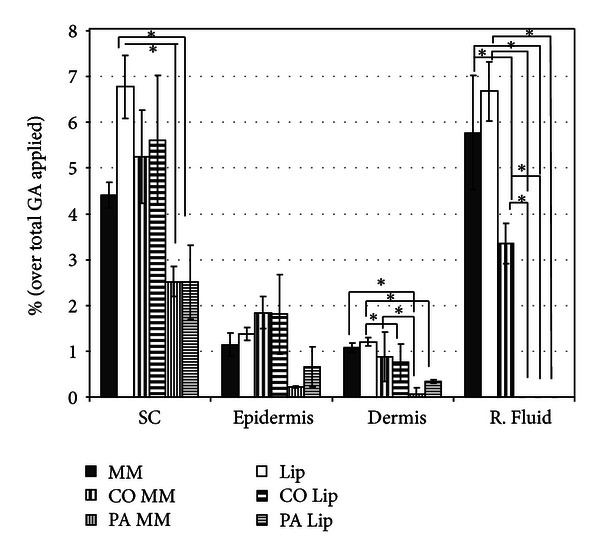
*In vitro* percutaneous absorption of gallic acid (GA) in Lip and MM formulations and the PA and CO cosmetotextiles (SC: stratum corneum, R. Fluid: receptor fluid) (significant level accepted *P < 0.01).

The aim of this assay was to demonstrate tracer delivery into the different layers of the skin. GAs encapsulated in MMs and Lips, which were either embedded or not embedded in cosmetotextiles, were applied to the skin to study the percutaneous absorption profiles of the agents. The GA extracted from a washing sample, the fabric, the stratum corneum, the rest of the epidermis, the dermis, and the receptor fluid was analysed. The results are listed in Table 4 and graphically represented in Figure 2.

Comparison of percutaneous absorption in percentage indicates that it is higher when GA was applied as a formulation (Lip or MM) than when it is applied through cosmetotextile. Besides, CO delivers to the skin GA in a greater extent than PA.

As shown in Figure 2, the penetration of GA formulated in Lip was much higher than that of GA formulated in MM. All skin compartments showed a higher amount of GA when vehiculised with Lip than when vehiculised with MM. This result could be due to the bilayer structure of the Lip, which is similar to the lipid bilayer structures present in the SC and in the cellular membranes of the skin [28]. Evidence that Lips do not penetrate deeper than the stratum corneum layer has been published [29]. However, Lips enhance the penetration of both hydrophilic and lipophilic drugs [30, 31]. However, both formulations promote the incorporation of a significant amount of GA in all skin layers, as indicated by the outstanding amount detected in the receptor fluid, which accounts for the amount that would be present at a systemic level.

Significant differences at a high level of *P < 0.01 can be visualized in Figure 2. Therefore, besides all previous comments, it is important to remark the significant difference between the amount of GA in the SC when applied Lip related to its corresponding PA textile and between the two formulations and their corresponding textiles in the deep layers of the skin (dermis and receptor fluid).

When GA was embedded into the cosmetotextiles, a marked reservoir effect was always induced. A lower degree of GA skin penetration was obtained in most skin layers compared with the results obtained for the formulation application alone. A similar penetration profile was obtained for the textiles treated with GA in MM or Lip in the skin compartments, SC, epidermis, and even in the dermis. In the dry textiles, the different lipid structures of Lip and MM, which may induce different enhancement behaviours, were lost. Therefore, textiles embedded with different vehicles may be expected to play a similar reservoir role. It is important to note that GA was absent in the receptor fluid of both the Lip-treated textiles and MM-treated PA. GA was only detected in the MM-treated CO fabric and in a smaller percentage than that detected with the free formulations. 

A comparison between the two textiles shows that much higher global percutaneous absorption was observed in the CO than in the PA fabric. GA was present in greater amounts in all skin layers when CO cosmetotextiles were topically applied and even reached the receptor fluid when applied through MM. As in the washing desorption process, it seems that PA has greater substantivity for GA than CO vehiculised either in MM or Lip, because lecithin has been reported to incorporate mainly on the surface of CO fibres, whereas interaction with PA occurs to a greater extent in the interior [27]. Therefore, it seems reasonable to predict a higher reservoir effect for PA textiles, promoting a lower percutaneous absorption of GA.

## 4. Conclusions

The precise amount of active agents present in cosmetotextiles was determined before being used in a textile drug delivery system. Much greater absorption of the formulations was found for the MM treatments relative to that observed for the Lip treatments. However, the MM-treated fabrics showed much higher desorption, leading to a lower amount of absorbed material in the textile after washing. A large increase in particle size from 7 to 200 nm was observed for MM, which indicates Lip formation due to surfactant dialysis. However, this increment in size does not help the formulation remain in the textile; on the contrary, it favours the desorption of the formulation. 

The percutaneous absorption of two formulations, Lip and MM, was evaluated, as was that of CO and PA textiles impregnated with the same Lip or MM. The results indicate that the penetration of GA formulated as Lip is much higher than that when formulated as MM. The bilayer structure of the vesicles, which is similar to the lipid bilayer structures present in the stratum corneum and in the rest of the skin, may account for their affinity.

When GA was embedded in the cosmetotextiles, it always promoted a reservoir effect, especially in the case of the PA fabrics. A similar penetration profile was obtained for the textiles treated with GA in MM or Lip in the different skin compartments. GA was absent in the receptor fluid of both Lip-treated textiles and in the MM-treated PA; it was only detected in the MM-treated CO fabric and in a smaller amount than that in the free formulations. This methodology may be useful to verify the penetration through human skin of encapsulated substances applied to textile materials, which can be considered as strategic delivery systems for the release of a given active principle at specific doses in the skin.

## Figures and Tables

**Table 1:** Mean size and polydispersity index (PDI) of initial liposome (Lip) and mixed micelle (MM) formulations and their dilutions in water.

Formulation	Mean size (nm)	PDI
Lip	717.40 ± 56.25	0.74 ± 0.03
10% Lip in water	407.47 ± 24.07	0.79 ± 0.03
5% Lip in water	367.80 ± 8.51	0.84 ± 0.10
2.5% Lip in water	395.07 ± 28.84	0.60 ± 0.14
MM	8.05 ± 0.08	0.13 ± 0.02
10% MM in water	8.23 ± 0.17	0.10 ± 0.01
5% MM in water	10.53 ± 0.06	0.14 ± 0.05
2.5% MM in water	55.35 ± 0.08	0.09 ± 0.01

**Table 2:** Percentage of formulation remaining in PA and CO fabrics after treatment, after first water washing, and after total water washings.

Fabric	Type of treatment	Treatment (% owf)	After 10 mL wash (% owf)	After total wash (% owf)
CO	Lip	10.99 ± 0.39	7.32 ± 2.43	5.58 ± 0.39
CO	MM	35.43 ± 2.73	12.72 ± 0.44	2.42 ± 0.06
PA	Lip	16.34 ± 1.23	12.73 ± 1.94	7.31 ± 0.64
PA	MM	40.14 ± 4.23	18.29 ± 1.98	3.91 ± 0.11

% owf: % over weight of fibre.

**Table 3:** Size (*Z*-average) and polydispersity index (PdI) of different baths of CO and PA subjected to bath exhaustion with Lip or MM.

Treatment	Analysed bath	Size (*Z*-average) diameter (nm)	PdI
CO/Lip	Initial bath	525.6 ± 26.1	0.68 ± 0.03
	Bath after exhaustion treatment	375.3 ± 64.6	0.51 ± 0.12
	Bath after 1st water washing (10 mL)	474.3 ± 32.2	0.68 ± 0.20
	Bath after 3rd water washing (50 mL)	623.5 ± 18.8	0.52 ± 0.03

CO/MM	Initial bath	6.9 ± 0.8	0.98 ± 0.04
	Bath after exhaustion treatment	102.2 ± 30.9	0.98 ± 0.03
	Bath after 1st water washing (10 mL)	206.7 ± 76.5	0.34 ± 0.12
	Bath after 3rd water washing (50 mL)	211.0 ± 38.2	0.31 ± 0.02

PA/Lip	Initial bath	525.6 ± 26.0	0.68 ± 0.03
	Bath after exhaustion treatment	460.6 ± 76.4	0.45 ± 0.01
	Bath after 1st water washing (10 mL)	510.0 ± 53.2	0.88 ± 0.21
	Bath after 3rd water washing (50 mL)	660.3 ± 31.9	0.49 ± 0.04

PA/MM	Initial bath	6.6 ± 0.8	0.97 ± 0.04
	Bath after exhaustion treatment	157.2 ± 81.7	0.62 ± 0.17
	Bath after 1st water washing (10 mL)	257.6 ± 97.9	0.05 ± 0.12
	Bath after 3rd water washing (50 mL)	166.9 ± 89.5	0.34 ± 0.07

**Table:** (a)

GA in formulations	GA in Lip		GA in MM	
Compartments	%	*µ*g/cm²	%	*µ*g/cm²
Total applied	—	**76.34**	—	**79.03**
Wash/fabric	83.97 ± 0.40	55.08 ± 1.40	87.61 ± 1.87	59.38 ± 2.33
SC	6.78 ± 0.69	4.46 ± 0.55	4.41 ± 0.28	2.98 ± 0.14
E	1.37 ± 0.14	0.90 ± 0.07	1.14 ± 0.25	0.77 ± 0.15
D	1.20 ± 0.09	0.78 ± 0.04	1.07 ± 0.10	0.73 ± 0.05
R. F	6.68 ± 0.65	4.38 ± 0.36	5.77 ± 1.25	3.90 ± 0.77
Perc. Abs (E + D + RF)	9.25 ± 0.86	6.06 ± 0.48	7.98 ± 1.60	5.40 ± 0.9

Recovery		65.59 ± 1.65		67.76 ± 1.23

**Table:** (b)

GA in CO	GA in Lip on CO		GA in MM on CO	
Compartments	%	*µ*g/cm²	%	*µ*g/cm²
Total applied	—	**147.95**	—	**242.61**
Wash/fabric	91.84 ± 9.72	131.28 ± 14.38	87.34 ± 6.30	210.85 ± 15.68
SC	5.60 ± 1.42	8.60 ± 2.26	5.24 ± 1.02	12.64 ± 2.48
E	1.81 ± 0.87	2.78 ± 1.93	1.84 ± 0.35	4.45 ± 0.86
D	0.75 ± 0.41	1.15 ± 0.66	0.87 ± 0.54	2.12 ± 1.30
R. F	0.00 ± 0.00	0.00 ± 0.00	4.72 ± 0.44	16.10 ± 1.03
Perc. Abs (E + D + RF)	2.56 ± 0.92	3.93 ± 1.99	7.43 ± 0.51	22.67 ± 1.22

Recovery	—	143.81 ± 4.25	—	241.47 ± 11.69

**Table:** (c)

GA in PA	GA in Lip on PA		GA in MM on PA	
Compartments	%	*µ*g/cm²	%	*µ*g/cm²
Total applied	—	**395.90**	—	**479.59**
Wash/fabric	96.64 ± 5.56	403.30 ± 22.02	97.20 ± 6.21	464.15 ± 29.78
SC	2.51 ± 0.81	10.49 ± 3.21	2.52 ± 0.33	12.05 ± 1.57
E	0.66 ± 0.44	2.78 ± 1.76	0.22 ± 0.02	1.05 ± 0.08
D	0.33 ± 0.04	1.39 ± 0.16	0.06 ± 0.10	0.28 ± 0.49
R. F	0.00 ± 0.00	0.00 ± 0.00	0.00 ± 0.00	0.00 ± 0.00
Perc. Abs (E + D + RF)	0.99 ± 0.55	4.17 ± 1.85	0.28 ± 0.03	1.33 ± 0.09

Recovery	—	417.75 ± 31.87	—	477.53 ± 27.71

SC: stratum corneum, E: epidermis, D: dermis, and R.F: receptor fluid.
